# Measurement site of inferior vena cava diameter affects the accuracy with which fluid responsiveness can be predicted in spontaneously breathing patients: a post hoc analysis of two prospective cohorts

**DOI:** 10.1186/s13613-020-00786-1

**Published:** 2020-12-11

**Authors:** Morgan Caplan, Arthur Durand, Perrine Bortolotti, Delphine Colling, Julien Goutay, Thibault Duburcq, Elodie Drumez, Anahita Rouze, Saad Nseir, Michael Howsam, Thierry Onimus, Raphael Favory, Sebastien Preau

**Affiliations:** 1 Division of Intensive Care, University of Lille, CHU Lille, 59000 Lille, France; 2 Department of Biostatistics, BST. University of Lille, ULR 2694 - METRICS : Évaluation des Technologies de Santé et des Pratiques Méd icales, CHU Lille, 59000 Lille, France; 3 University of Lille, Inserm, Institut Pasteur de Lille, U1167, 59000 Lille, France; 4 Division of Intensive Care, University of Lille, CHU Lille, Inserm, Institut Pasteur de Lille, Hôpital Roger Salengro, U1167 Avenue Emile Laine, 59000 Lille, France

**Keywords:** Inferior vena cava, Fluids, Fluid responsiveness, Hemodynamic, Sepsis, Severe infection, Ultrasound, Echocardiography, Collapsibility index, Spontaneous breathing

## Abstract

**Background:**

The collapsibility index of the inferior vena cava (cIVC) has potential for predicting fluid responsiveness in spontaneously breathing patients, but a standardized approach for measuring the inferior vena cava diameter has yet to be established.

The aim was to test the accuracy of different measurement sites of inferior vena cava diameter to predict fluid responsiveness in spontaneously breathing patients with sepsis-related circulatory failure and examine the influence of a standardized breathing manoeuvre.

**Results:**

Among the 81 patients included in the study, the median Simplified Acute Physiologic Score II was 34 (24; 42). Sepsis was of pulmonary origin in 49 patients (60%). Median volume expansion during the 24 h prior to study inclusion was 1000 mL (0; 2000). Patients were not severely ill: none were intubated, only 20% were on vasopressors, and all were apparently able to perform a standardized breathing exercise. Forty-one (51%) patients were responders to volume expansion (i.e. *a* ≥ 10% stroke volume index increase). The cIVC was calculated during non-standardized (cIVC-ns) and standardized breathing (cIVC-st) conditions. The accuracy with which both cIVC-ns and cIVC-st predicted fluid responsiveness differed significantly by measurement site (interaction *p* < 0.001 and < 0.0001, respectively). Measuring inferior vena cava diameters 4 cm caudal to the right atrium predicted fluid responsiveness with the best accuracy. At this site, a standardized breathing manoeuvre also significantly improved predictive power: areas under ROC curves [mean and (95% CI)] for cIVC-ns = 0.85 [0.78–0.94] versus cIVC-st = 0.98 [0.97–1.0], *p* < 0.001. When cIVC-ns is superior or equal to 33%, fluid responsiveness is predicted with a sensitivity of 66% and a specificity of 92%. When cIVC-st is superior or equal to 44%, fluid responsiveness is predicted with a sensitivity of 93% and a specificity of 98%.

**Conclusion:**

The accuracy with which cIVC measurements predict fluid responsiveness in spontaneously breathing patients depends on both the measurement site of inferior vena cava diameters and the breathing regime. Measuring inferior vena cava diameters during a standardized inhalation manoeuvre at 4 cm caudal to the right atrium seems to be the method by which to obtain cIVC measurements best-able to predict patients’ response to volume expansion.

## Background

Correcting acute reductions in blood volume through volume expansion (VE) is essential, but excessive VE can increase morbidity and mortality in critically ill patients [1–5]. Despite the importance of accurately predicting the response to VE in cases of acute circulatory failure, its clinical assessment is erroneous in 50% of cases [6–10]. Dynamic parameters have been proposed to predict fluid responsiveness (FR) in critically ill patients [11–13]. Initially developed for mechanically ventilated patients, use of these parameters has been proposed in spontaneously breathing (SB) patients in an effort to avoid unnecessary fluid exposure [14, 15]. Unfortunately, most of these dynamic parameters are unsuitable for SB patients. In fact, under spontaneous breathing, respiratory changes in the stroke volume index (SVI) or their estimates (e.g. respiratory changes in arterial pulse pressure) are either inaccurate as predictors of FR [16], or too complex for routine clinical use [17, 18]. Conversely, passive leg raising-induced change in SVI is an accurate predictor, which has been validated in SB patients [19, 20]; but it requires a tool to measure (or estimate) cardiac output or stroke volume, and may be either technically impossible or unreliable under specific conditions (e.g. insufficient increase in central venous pressure during the procedure [21], pregnancy [22] or intra-abdominal hypertension [23, 24]).

Measuring the diameter of the inferior vena cava (IVC) by ultrasonography is a fast, non-invasive tool for assessment of the blood volumetric status of critically ill patients in the early stages of care [25–27]. In SB patients, and in the absence of a standardized respiratory manoeuvre during ultrasound recordings, IVC collapsibility (cIVC) predicts FR with high specificity but low sensitivity [28–33]. Reversible, non-invasive procedures that magnify the change in intrathoracic and transpulmonary pressure (e.g. a deep inhalation or Valsalva manoeuvre) can improve the accuracy of some parameters to predict FR [17, 18]. In two recent studies, Preau et al*.* and Bortolotti et al*.* showed that a standardized, deep inhalation improved the sensitivity of cIVC to predict FR without altering its specificity [31, 32]. The use of cIVC to predict FR is scarce, but has been described in current practice [25–27] and controlled, trial protocols [34, 35]. Different measurement sites have been proposed to measure cIVC, ranging from 0.5 to 4 cm caudal to the IVC–right atrium junction [28–32]. It has been demonstrated that the collapsibility and distensibility of the IVC are both affected by the segment of vein considered [36, 37]. However, a rigorous comparison of the accuracy with which different sites for measuring IVC diameter can predict FR has yet to be published. This study aimed to analyse the effect of the measurement site, and the use of a concomitant, standardized breathing manoeuvre, on the accuracy with which cIVC measurements predict responsiveness to VE in SB patients with sepsis.

## Methods

### Study design

The present study was conducted post hoc on the ultrasound analyses of all patients included at the Lille University Hospital from two prospective cohorts [31, 32]. The initial objective of these cohorts was to investigate whether the cIVC, measured without (cIVC-ns) or with a standardized inhalation (cIVC-st), predicts FR in septic patients. The first study cohort focused on consecutive patients with regular sinus rhythm and included 90 patients from November 2011 to January 2014 [31]. The second, of identical design, looked at 55 consecutive patients who presented with cardiac arrhythmia (atrial fibrillation or more than 6 extrasystoles per minute) for which the inclusions ran from May 2012 to May 2015 [32].

### Patients

Inclusion criteria were as follows: SB, adult patients admitted with sepsis in five intensive care units of the Lille University Hospital [38] with one or more clinical signs of acute circulatory failure for which a physician has prescribed VE. Clinical signs of acute circulatory failure were defined as systemic arterial hypotension (systolic arterial pressure < 90 mm Hg, or a decrease > 40 mm Hg in previously hypertensive patients), oliguria (urine output < 0.5 mL/kg/h over 1 h or more), tachycardia (heart rate > 100 per min), or mottled skin. Exclusion criteria included high-grade aortic insufficiency, transthoracic echogenicity unsuitable for measuring the velocity–time integral of aortic blood flow or IVC diameter, clinical signs of active exhalation, clinical or ultrasonographic evidence of pulmonary oedema due to heart failure [39], pregnancy, or abdominal compartment syndrome [40]. Patients for whom we could not measure IVC diameters in the first 4 cm were also excluded.

### Study protocol

Ultrasonographic and clinical data were recorded prospectively immediately before and after VE, which consisted of a 30-min infusion of 500 mL 4% gelatin (Gelofusine® 4%, B. Braun, Melsungen, Germany or Plasmion®, Fresenius-Kabi, Louviers, France). Ultrasound examination was performed in a semi-recumbent position (30–45°) using commercially available Vivid-i and Vivid-S5 (General Electric, Solingen, Germany) with a phased array, low-frequency (2 MHz) transducer, operated by trained physicians (Level 2–3) [41]. The velocity–time integral of aortic blood flow was measured by pulsed-wave Doppler on a five-chamber apical view during unstandardized, spontaneous breathing [42]. The IVC bi-dimensional records were generated using the subcostal long-axis view. Particular attention was made to obtain a satisfactorily long axis section of the vessel at its centre in the sagittal plane. The ultrasound recordings of IVC were carried out over 3 consecutive breathing cycles (without and with a standardized breathing manoeuvre), both before and immediately after VE. The standardized breathing manoeuvre consisted of a brief (< 5 s) and continuous inhalation effort. Patients were verbally coached to generate a minimum buccal pressure from − 5 to − 10 mm H_2_O without any respiratory resistor. Oral cavity pressures were recorded with commercially available MP101 micromanometers (KIMO Instrument, Montpon, France), connected via a plastic sampling line to an antibacterial filter in series with a S183 mouth end piece (Teleflex Medical, Int’Air medical, Bourg-en-Bresse, France), as previously described [31, 32].

**Fig. 1:**
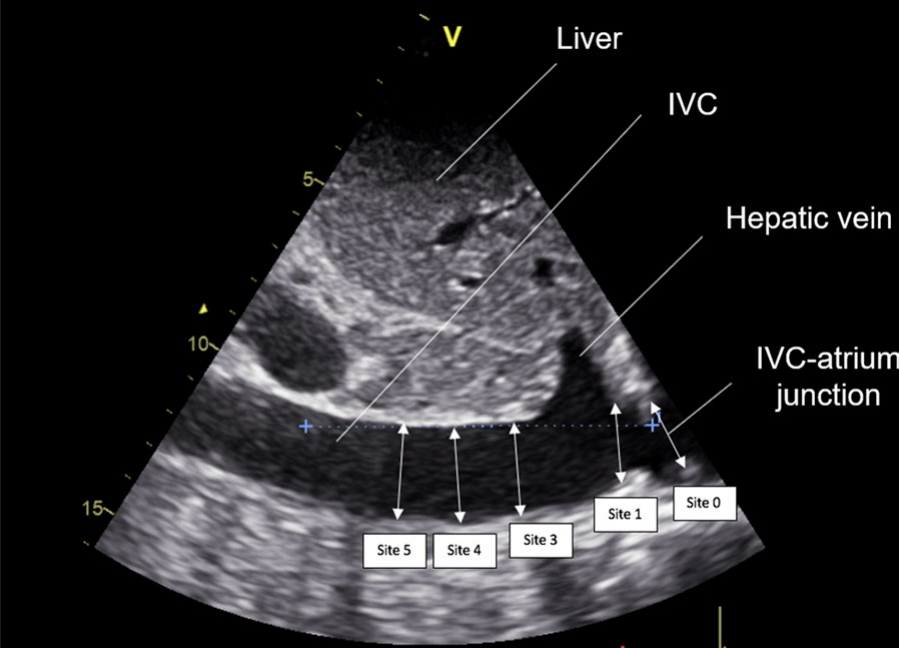
Bi-dimensional ultrasound recording of the inferior vena cava (IVC) generated using the sub-costal, long-axis view. Measurement of IVC diameters were carried out at five sites: at the IVC–right atrium junction (site 0), then at 1 (site 1), 3 (site 3), 4 (site 4) and 5 cm (site 5) caudal to the IVC–atrial junction

The anonymized recordings were analysed remotely. The SVI and general echocardiographic measurements were performed by echocardiography-trained, intensive care staff who were blind to both the clinical data and IVC measurements. To calculate the SVI, the left ventricular outflow tract (LVOT) velocity–time integral (VTI) was averaged over 15 consecutive cardiac cycles. The SVI equalled the product of the LVOT VTI multiplied by the ratio of the aortic valve area to the body surface area [42]. The aortic valve area was calculated once from the average aortic annulus diameter over three measurements. The body surface area calculated once according to the DuBois Method [43], was considered constant. Relative changes in SVI induced by VE were expressed as a percentage: VE-induced change in SVI (%) = 100 x ((post-VE value−baseline value) / baseline value). Patients were considered to have responded to treatment if VE induced a change in SVI of > 10% (i.e. VE-induced > 10% change in the LVOT VTI). This method is accepted as the reference method to assess the response to VE in SB patients [44, 45]. Moreover, this threshold value appeared to be clinically relevant (i.e. in terms of VE-induced changes in systolic arterial pressure and pulse pressure) and was also at least twice the intra-observer variability in the LVOT VTI measured in our previous studies [29, 30]. The IVC measurements were carried out using EchoPAC PC Software (General Electric Healthcare, Chicago, USA) by different operators, blind to the SVI and general echocardiographic measurements. The measurements of IVC diameters on both inhalation and exhalation were carried out at 5 separate sites: the cavo-atrial junction (site 0), then at 1, 3, 4 and 5 cm (sites 1, 3, 4 and 5, respectively) caudal to this junction, under both unstandardized and standardized breathing conditions (Fig. 1). Measurements of all IVC diameters were realized consecutively from site 0 to 5 for a given patient. The site at 2 cm was excluded a priori in view of its poor reproducibility owing to the entry at this site of the hepatic vein. Diameters were measured perpendicular to IVC walls from the trailing edge to the leading edge of the anterior and posterior wall, respectively. Distances between anatomical levels of the IVC and the cavo-atrial junction were measured parallel to IVC walls. The mean of three measurements was used to calculate the IVC collapsibility index (cIVC) as: ((maximum expiratory diameter—minimum inspiratory diameter) × 100/expiratory diameter).

### Statistical analysis

Categorical variables are expressed as counts and percentages. Quantitative variables are reported as medians with interquartile ranges (25th; 75th centiles). Normality of distributions was assessed using histograms and the Shapiro–Wilk test. The use of norepinephrine was compared between responders and non-responders with the Chi-square test. Comparison of the cIVC according to FR and the measurement site (without and with a standardized breathing manoeuvre) were assessed by linear mixed models including FR, measurement site, interaction between FR and measurement site as fixed effects, and the patient as a random effect (in order to take repeated measurements into account). Normality of residuals was checked by plotting QQ-plot of conditional studentized residuals. When the interaction between FR and measurement site was significant, the cIVC according to FR was compared by measurement site using linear contrast applying Bonferroni correction. For each measurement site where a significant result was found, the areas under the receiver operating characteristic curves (ROC) and their 95% confidence intervals (CIs) were calculated. The optimal threshold values were determined by maximizing the Youden Index. Sensitivity and specificity for these values were calculated with their 95% CIs. We also report the threshold values to reach a sensitivity or specificity ≥ 0.90. The correlation between the cIVC and the variation of SVI after VE was assessed with the Spearman’s correlation coefficient. Statistical testing was performed at the two-tailed α level of 0.05. Data were analysed using the SAS software package, release 9.4 (SAS Institute, Cary, NC).

## Results

Among the 145 patients from the two prospective cohorts [31, 32], 95 were included at the Lille University Hospital. Among them, 14 were excluded: 2 for unusable corrupted files and 12 for recordings that did not permit all IVC measurements to be taken from site 1 to 4. Eventually, 81 patients with a median age of 64 years old (53; 74) were included. The general characteristics of studied patients are summarized in Table 1 [46].

**Table 1:** General characteristics of the patients at inclusion

General data	Total population	Non-responders	Responders
	(*n* = 81)	(*n* = 40)	(*n* = 41)
Age (year)	64 (54; 73)	63 (51; 76)	64 (55; 72)
Sex ratio female/male	27/54	15/25	12/29
Height (m)	1.71 (1.65; 1.76)	1.69 (1.63; 1.75)	1.72 (1.65; 1.80)
Weight (kg)	73 (60; 89)	70 (60; 85)	77.5 (60; 90)
SAPS II	34 (24; 42)	37 (28; 48)	31 (22; 39)
Pre-existing conditions			
Arterial hypertension	31 (38)	17 (43)	14 (34)
Chronic left ventricular failure	8 (10)	4 (10)	4 (10)
Chronic right ventricular failure	3 (4)	2 (5)	1 (2)
Atrial fibrillation	18 (22)	8 (20)	10 (24)
COPD	17 (21)	9 (23)	8 (20)
Chronic pulmonary hypertension	6 (7)	5 (13)	1 (2)
Pulmonary embolism	2 (3)	0 (0)	2 (5)
Arteriopathy	9 (11)	7 (18)	2 (5)
Sepsis			
Pulmonary	49 (61)	24 (60)	25 (61)
Skin and soft tissue	13 (16)	7 (18)	6 (15)
Abdominal	10 (12)	5 (13)	5 (12)
Urinary	6 (7)	3 (8)	3 (8)
Other infections	3 (4)	1 (3)	2 (5)
Hemodynamic parameters			
Mean arterial pressure (mm Hg)	68 (61; 79)	67(61; 77)	70 (61; 83)
Heart rate (beats/min)	107 (88; 120)	98 (83; 114)	109 (102; 126)
LVOT VTI (cm)	16.4 (13.2; 19.7)	17.4 (15.2; 21)	14.8 (12; 18.4)
Cardiac index (L/min/m^2^)	2.9 (2.3; 3.7)	2.9 (2.5; 4.1)	2.9 (2.2; 3.3)
Norepinephrine	16 (20)	13 (33)	3 (7)
Norepinephrine µg/kg/min	0.23 (0.17; 0.36)	0.25 (0.14; 0.35)	0.22 (0.19; 0.37)
VE in last 24 h (mL)	1000 (0; 2000)	1000 (0; 2000)	1000 (30; 1700)
Oliguria	39 (48)	18 (45)	21 (51)
Arterial hypotension	38 (47)	19 (48)	19 (46)
Tachycardia	57 (70)	24 (60)	33 (80)
Mottled skin	23 (28)	15 (38)	8 (20)

Values are expressed as counts (%) or medians and interquartile ranges (25th; 75th centiles). *COPD* chronic obstructive pulmonary disease, *SAPS* Simplify Acute Physiology Score, *LVOT VTI* left ventricular outflow tract velocity time integral, *VE* volume expansion

Among the population, 37 patients (45%) had cardiac arrhythmias, and the median left ventricular ejection fraction before VE was 62% (57; 67). The median inspiratory depression generated without a standardized breathing manoeuvre was lower than when patients were verbally encouraged to perform a standardized inhalation (− 0.5 mm H_2_O (− 1; 0) and -6 mm H_2_O (− 7; − 5), *p* < 0.0001). Intra-patient variability regarding unstandardized and standardized inspiratory depression was 0.4 mm H_2_O (0; 0.4) and 1.8 mm H_2_O (1.3; 2.5), respectively. The time needed to perform both IVC recordings (online) and cIVC measurements (blinded, offline) was not assessed in the present study. Intra- and inter-observer variability with regards to the velocity–time integral of aortic blood flow was 3.5% (1.6; 5.4) and 7.9% (5.4; 10.3), respectively. Intra- and interobserver cIVC-st variability was 8.6% (4.4; 12.9) and 9.7% (2.7;17.3), respectively.

**Fig. 2:**
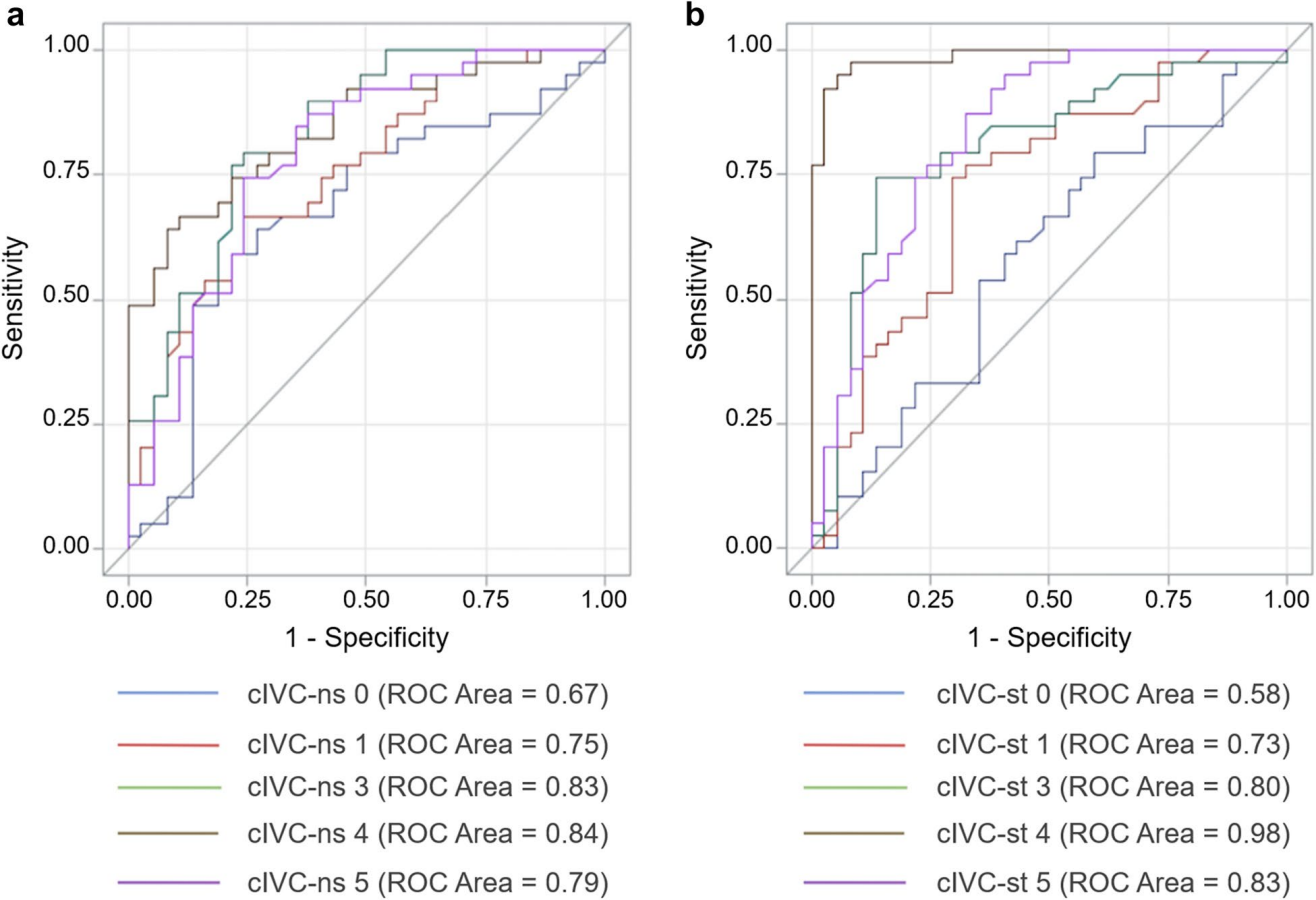
Area under the receiver operating characteristic (ROC) curves to predict fluid responsiveness**.** Area under the ROC curves of the collapsibility index of the inferior vena cava (cIVC) to predict fluid responsiveness depending on the measurement site without (**a**, cIVC-ns), and with (**b**, cIVC-st) a standardized breathing manoeuvre. Measurement of inferior vena cava (IVC) diameters was carried out at five sites: at the IVC–right atrium junction (site 0), then at 1 (site 1), 3 (site 3), 4 (site 4) and 5 cm (site 5) caudal to the IVC–atrial junction. For each site where a difference was found between responders and non-responders, the areas under the ROC curves were calculated

**Fig. 3:**
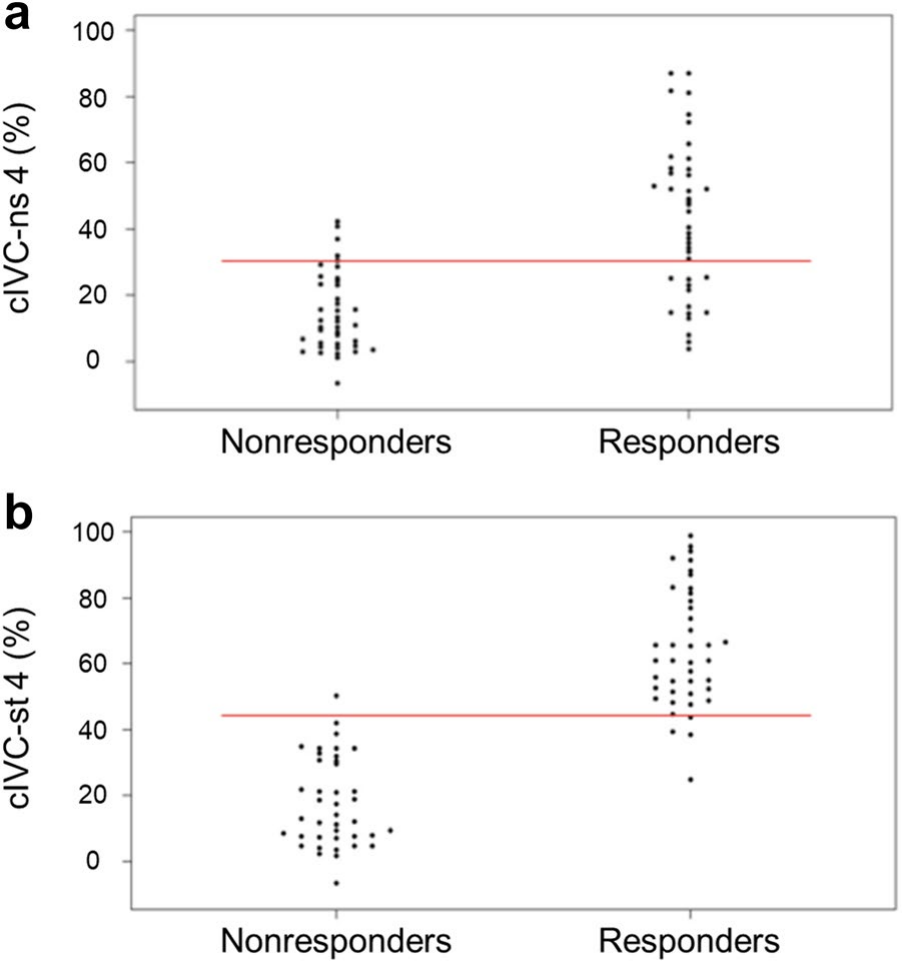
Scatterplot of individual values for the collapsibility index of the inferior vena cava (cIVC). Scatterplot of individual values before volume expansion (VE) for cIVC measured at 4 cm caudal to the IVC–atrial junction in spontaneous breathing patients, both without (**a**, cIVC-ns 4) and with (**b**, cIVC-st 4) standardized breathing manoeuvre, in non-responders and responders to VE. Red lines show the optimal threshold value for predicting fluid response

For the whole group, VE increased the SVI by 10% (1; 27). Distribution of VE increased in SVI (i.e. VE-induced change in the LVOT VTI) was not normal (*p* = 0 < 0.001), and patients were divided as follows: 14 with a VE-increase in SVI between -20 and -0.1%, 36 between 0 and 19.9%, 17 between 20 and 39.9%, and 14 between 40 and 59.9%. Forty-one patients (51%) were responders to VE. The VE-induced increase in SVI was 1% (-2; 4) in non-responders and 27% (20; 42) in responders. Twenty-six (65%) non-responders had a VE-related change in LVOT VTI between 0 and 10%, and 27 (66%) responders had a VE-related change in LVOT VTI between 10 and 30%. The general characteristics of the non-responder and responder patients are summarized in Table 1. Non-responders were more likely to receive norepinephrine than responders (33% versus 7%, *p* = 0.004). The cIVC for each measurement site is presented in Table 2 for non-responders and responders. The relationship between cIVC and FR differed significantly depending on the measurement site in both unstandardized (interaction *p* = 0.0005) and standardized (interaction *p* < 0.0001) breathing conditions. For cIVC-ns, no difference was found between responders and non-responders for site 0 (*p* = 0.086), but a significant difference was found for all other sites (*p* < 0.0001 for all). With a standardized inhalation manoeuvre, no difference in cIVC-st was found between responders and non-responders at the IVC–right atrium junction (site 0) (*p* = 0.89), but there was a significant difference at all the other measurement sites: 1 cm (*p* = 0.0010) and 3, 4 and 5 cm (*p* < 0.0001). For each site where a difference was found between responders and non-responders, the areas under the ROC curves were calculated (Fig. 2), and optimal thresholds to predict FR are presented in Table 3. Thresholds for a sensitivity or a specificity of 0.9 are given for measurement sites 1, 3, 4 and 5 in Additional file 1. Individual values of cIVC-ns and cIVC-st measured at 4 cm caudal to the right atrium are given in Fig. 3. The accuracy with which cIVC-st measurements at this site predicted FR was significantly better than cIVC-ns, with respective area under ROC curve values of 0.98 [0.97; 1] and 0.85 [0.78; 0.94] (*p* = 0.0008).

**Table 2:** Collapsibility index of the inferior vena cava (cIVC) in non-responders and responders before volume expansion

Breathing	Measurement	Non-responders	Responders
Condition	Site	(*n* = 40)	(*n* = 41)
cIVC-ns (%)	0	19 (8; 28)	35 (20; 44)
	1	22(10; 34)	41 (24; 53)
	3	11 (3; 18)	36 (20; 67)
	4	12 (5; 24)	45 (25; 58)
	5*	7 (2; 18)	29 (17; 56)
cIVC-st (%)	0	32 (15; 47)	41 (28; 51)
	1	34 (19; 52)	54 (42; 65)
	3	26 (11; 42)	61 (49; 82)
	4	13 (8; 31)	61 (52; 79)
	5*	14 (3; 32)	55 (32; 72)

Values are expressed as counts (%) or medians and interquartile ranges (25th; 75th centiles) Measurements of inferior vena cava (IVC) diameters were carried out at five sites: at the IVC-atrium junction (site 0), then at 1 (site 1), 3 (site 3), 4 (site 4) and 5 cm (site 5) caudal to the IVC-atrium junction. *cIVC-ns* collapsibility index of the IVC in non-standardized breathing conditions, *cIVC-st* collapsibility index of IVC with standardized breathing manoeuvre. *Measurement site 5 was performed in only 37 non-responders and 39 responders

**Table 3:** Accuracy with which the collapsibility index of the inferior vena cava (cIVC) predicts fluid responsiveness

Breathing condition	Site	ROC [95% CI]	Optimal threshold (%)	Sensitivity	Specificity
cIVC-ns	1	0.76 [0.66; 0.86]	> 35*	0.68	0.78
	3	0.82 [0.74; 0.91]	> 20*	0.76	0.78
	4	0.85 [0.78; 0.94]	> 33*	0.66	0.92
	5	0.78 [0.68; 0.89]	> 19*	0.74	0.76
cIVC-st	1	0.73 [0.62; 0.84]	> 42*	0.76	0.66
	3	0.80 [0.71; 0.91]	> 49*	0.76	0.85
	4	0.98 [0.97; 1]	> 44*	0.93	0.98
	5	0.83 [0.74; 0.92]	> 25*	0.87	0.67

Measurements of inferior vena cava (IVC) diameters were carried out at five sites: at the IVC-atrium, then at 1 (site 1), 3 (site 3), 4 (site 4) and 5 cm (site 5) caudal to the IVC-atrium junction. *ROC* area under the curve of receiver operating characteristics, *CI* confidence interval, *cIVC-ns* collapsibility index of the IVC in non-standardized breathing conditions,* cIVC-st* collapsibility index of IVC with standardized breathing manoeuvre. *Optimal threshold value to predict response to volume expansion

There was a linear correlation between cIVC-st before VE and the VE-induced change in SVI (*r* = 0.82; *p* < 0.0001), and between cIVC-ns before VE and the VE-induced change in SVI (r = 0.62; *p* < 0.0001), at site 4 (Additional file 2).

## Discussion

The present study is the first, to our knowledge, to demonstrate that the relationship between cIVC and FR is significantly affected by the measurement site of IVC diameter. Our results confirm that clinicians should avoid measuring cIVC at site 0/1 [36], and suggests that the most reliable measurement site for predicting the cardiac response to VE is at 4 cm caudal (upstream) from the cavo-atrial junction. Moreover, this study is the first to demonstrate a significant impact of a standardized breathing manoeuvre on the accuracy with which cIVC predicts FR. In practical terms, in SB patients, a reduction in IVC diameter of more than 33% during a non-standardized inspiration, measured at 4 cm caudal to the cavo-atrial junction, predicts FR with low sensitivity but great specificity. Thus, in cases of low cIVC values during non-standardized inhalation (cIVC-ns), the response to VE is uncertain and use of a standardized breathing manoeuvre (cIVC-st) is to be recommended. In these cases, a cIVC-st of > 44% predicted FR with both high sensitivity and specificity when IVC diameters were measured 4 cm caudal to the right atrium.

Our study demonstrates that when IVC diameters are measured too close to the right atrium (i.e. ≤ 1 cm), cIVC predicts FR with only low specificity. This phenomenon was accentuated by a deep inhalation breathing manoeuvre. This may be because the diaphragm’s movement generated false-positives in proximal measurement sites, as suggested by Gignon et al*.* in a physiological study with healthy volunteers [47]. Nevertheless, this motion appears to have less impact at more distal measurement sites. Bortolotti et al*.* demonstrated that a deep inhalation did not impact cIVC in non-responders to VE when IVC diameters were measured beyond 3 cm caudal to the right atrium [32]. Most of the studies that analysed the accuracy of cIVC-ns to predict FR in SB patients assessed IVC diameters approximately 2–4 cm caudal to the right atrium [30–32]. Accordingly, these studies demonstrated that cIVC-ns predicted FR with good specificity, ranging from 88 to 97%. Moreover, when cIVC was measured between 3 and 4 cm caudal to the IVC–right atrium junction, a deep inhalation manoeuvre did not alter this specificity [31, 32]. Nonetheless, Lanspa et al*.* managed to predict FR with a specificity of 89% while measuring cIVC during gentle, spontaneous breathing conditions at distances of 0.5–3 cm from the right atrium [29]. The specificity of cIVC in predicting FR may have been preserved in their study by the calmness of the inhalation manoeuvre, which reduced the influence of diaphragm movement on the IVC diameter. To prevent poor specificity, therefore, measurements of cIVC ≤ 1 cm caudal to the right atrium should be avoided for the prediction of FR in SB patients, particularly when a deep inhalation manoeuvre is used. Our results confirm the conclusions from Wallace et al. that clinicians should avoid measuring cIVC at site 0/1 [36].

Our study nevertheless shows that cIVC-ns predicts FR with low sensitivity at all measurement sites of IVC diameter between 0 and 5 cm caudal to the right atrium. Moreover, this study is the first to demonstrate that a simple, standardized, deep inhalation manoeuvre significantly improves the sensitivity of cIVC measurements to predict FR, in particular when IVC diameters are measured beyond 3 cm caudal to the right atrium junction. The fact that cIVC-st gradually increases upstream from the junction of the vessel with the right atrium is counterintuitive physiologically. Indeed, the downstream segments of the IVC should be the most influenced by negative thoracic pressures during inspiration. The IVC can be characterized as a highly compliant, collapsible tube and variations in its diameter during the respiratory cycle can be explained by transmural pressure gradient variations [48, 49]. It is a large, elliptical, intra-abdominal vessel which becomes intra-thoracic in its last portion where it traverses the diaphragm through a fibrous anatomical orifice [50]. This anatomical feature was one of the impetuses for our study, because we suspected that the diaphragm may lower the sensitivity of cIVC in SB patients; the attachment of the diaphragm and the fibrous portion around the vessel may, we hypothesized, result in decreased collapsibility of its downstream segments. Accordingly, Wallace et al*.* showed, in spontaneously breathing volunteers, that cIVC was significantly lower when recorded closer to the right atrium (cIVC = 20%) than when recorded approximately 4 cm caudal to it (cIVC = 30%, *p* = 0.03), or at the left renal vein (cIVC = 30%, *p* = 0.002—approximately 5 cm caudal to the right atrium) [36]. Therefore, measurements ≤ 3 cm caudal to the right atrium should be avoided to prevent poor sensitivity of the prediction of FR in SB patients, even if a standardized breathing manoeuvre is employed.

Overall, our results support the clinical use of cIVC measurements in the sub-costal, long-axis view in the two-dimensional mode, perpendicular to the IVC wall at 4 cm caudal to the right atrium. The rigorous application of this technique guarantees the accuracy of IVC diameter measurements during the respiratory cycle and, may reduce the inter- and intra-observer variability of cIVC as demonstrated by Finnerty et al.[51].

Our study has several limitations. Firstly, this study was performed in a population of adult patients with a median body weight and height of 73 kg (60; 89) and 1.71 m (1.65; 1.76), respectively. Thus, the present results cannot necessarily be generalized to infants or adult patients of extreme size. Secondly, while the post hoc analysis design of two prospective cohorts does not permit us to determine the proportion for whom our preferred measurement site is exploitable, previous studies have demonstrated that IVC diameters could not be assessed in this region in 7% to 15% of SB patients with sepsis [31, 32]. Moreover, we cannot exclude that the admittedly impressive accuracy of cIVC-st (measured 4 cm caudal to the cavo-atrial junction) to predict FR might reflect a selection bias due to the post hoc and monocentric design of the study. Thirdly, IVC measurements were performed without systematic measurement of intra-abdominal pressure. We acknowledge that elevated intra-abdominal pressure may influence IVC diameters, and thus, may alter cIVC accuracy to predict FR [52]. Nevertheless, despite a possible improvement of cIVC diagnostic accuracy, a systematic measurement of bladder pressure would also increase recording complexity and alter measurement feasibility. The influence of intra-abdominal pressure on cIVC accuracy in predicting FR in SB patients has yet to be established. Fourthly, VE duration may have lasted up to 30-min in some patients. Thus, hemodynamic effects may be less marked or have waned at the time of the second echocardiography assessment in the corresponding patients. Eventually, the assessment of our dynamic variables was performed in a select population, since patients were only enrolled with infection-related acute circulatory failure, under none or only a low-dose of norepinephrine, and for whom VE has already been prescribed by the physician in charge. In the present study, non-responders were more likely to be on norepinephrine than responders, despite having received the same amount of fluid. As demonstrated before, the addition of vasopressors, through veno-constriction, increases the stressed volume and may be responsible for a lower prevalence of FR [53]. In addition, patients with active exhalation, abdominal compartment syndrome, pregnancy, obesity or abdominal surgery, all of which could interfere with cIVC accuracy, were excluded from the study. Overall, patients were not severely ill: none were intubated, only 20% were on vasopressors, and all were apparently able to perform a standardized breathing exercise. Overall, patients of the present study were not severely ill, and the results will be more applicable to physicians in emergency or intermediate care departments than in intensive care units. Therefore, our results require confirmation in an unselected, critically ill population.

## Conclusions

This rigorous comparison of different measurement sites suggests that the interaction between cIVC and FR is dependent on the way in which IVC diameter is measured: the best diagnostic accuracy for predicting FR is at 4 cm caudal to the cavo-atrial junction. We have also shown that a standardized, short (< 5 s) deep-inhalation manoeuvre improves the accuracy with which cIVC measurements predict patients’ response to VE. The cIVC thus measured is a fast, non-invasive and easy-to-implement tool which could improve clinical management of the acute phase of sepsis in SB patients. Its more widespread implementation in other critical conditions nevertheless requires a similarly rigorous, prospective study.

## Supplementary Information

**Additional file 1. **Accuracy with which the collapsibility index of the inferior vena cava (cIVC) predicts fluid responsiveness.

**Additional file 2.** Linear correlation between the collapsibility index of the inferior vena cava (cIVC) and fluid responsiveness.

## Data Availability

The datasets used and/or analysed during the current study are available from the corresponding author on reasonable request.

## Abbreviations

CI: Confidence Interval
cIVC: Collapsibility index of the Inferior Vena Cava
cIVC-ns: Collapsibility index of the Inferior Vena Cava during non-standardized breathing conditions
cIVC-st: Collapsibility index of the Inferior Vena Cava during a standardized breathing manoeuvre
COPD: Chronic obstructive pulmonary disease
FR: Fluid responsiveness
IVC: Inferior Vena Cava
LVOT: Left ventricular outflow tract
SAPS: Simplified Acute Physiology Score
SB: Spontaneously breathing
SVI: Stroke Volume Index
ROC: Receiver Operating Characteristic
VE: Volume expansion
VTI: Velocity–time integral
