# Supplementary material for: Measurement site of inferior vena cava diameter affects the accuracy with which fluid responsiveness can be predicted in spontaneously breathing patients: a post hoc analysis of two prospective cohorts
Source: Ann Intensive Care. 2020 Dec 11;10:168. doi: 10.1186/s13613-020-00786-1 (PMC7732956; doi:10.1186/s13613-020-00786-1)
Supplement: Supplementary file 1 — Additional file 1. Accuracy with which the collapsibility index of the inferior vena cava (cIVC) predicts fluid responsiveness. [file 13613_2020_786_MOESM1_ESM.docx]

**Additional file 1. Accuracy with which the collapsibility index of the inferior vena cava (cIVC) predicts fluid responsiveness**

| **Breathing condition** | **Site** | **Threshold (%)** | **Sensitivity** | **Specificity** |
| --- | --- | --- | --- | --- |
| **cIVC-ns** | 1 | > 35* | 0.68 | 0.78 |
|  |  | > 18 | 0.9 |  |
|  |  | > 49 |  | 0.9 |
|  | 3 | > 20* | 0.76 | 0.78 |
|  |  | > 14 | 0.9 |  |
|  |  | > 33 |  | 0.9 |
|  | 4 | > 33* | 0.66 | 0.92 |
|  |  | > 14 | 0.9 |  |
|  |  | > 31 |  | 0.9 |
|  | 5 | > 19* | 0.74 | 0.76 |
|  |  | > 9 | 0.9 |  |
|  |  | > 55 |  | 0.9 |
|  |  |  |  |  |
| **cIVC-st** | 1 | > 42* | 0.76 | 0.66 |
|  |  | > 25 | 0.9 |  |
|  |  | > 60 |  | 0.9 |
|  | 3 | > 49* | 0.76 | 0.85 |
|  |  | > 21 | 0.9 |  |
|  |  | > 61 |  | 0.9 |
|  | 4 | > 44* | 0.93 | 0.98 |
|  |  | > 35 | 0.9 |  |
|  |  | > 45 |  | 0.9 |
|  | 5 | > 25* | 0.87 | 0.67 |
|  |  | > 21 | 0.9 |  |
|  |  | > 60 |  | 0.9 |

*Measurements of inferior vena cava (IVC) diameters were carried out at five sites: at the IVC-atrium, then at 1 (site 1), 3 (site 3), 4 (site 4) and 5 cm (site 5) caudal to the IVC-atrium junction. ROC, area under the curve of Receiver Operating Characteristics; CI, Confidence Interval; cIVC-ns, collapsibility index of the IVC in non-standardized breathing conditions; cIVC-st, collapsibility index of IVC with standardized breathing manoeuvre. * Optimal threshold value to predict response to volume expansion.*
