# Supplementary figures and images for: Measurement site of inferior vena cava diameter affects the accuracy with which fluid responsiveness can be predicted in spontaneously breathing patients: a post hoc analysis of two prospective cohorts
Source: Ann Intensive Care. 2020 Dec 11;10:168. doi: 10.1186/s13613-020-00786-1 (PMC7732956; doi:10.1186/s13613-020-00786-1)

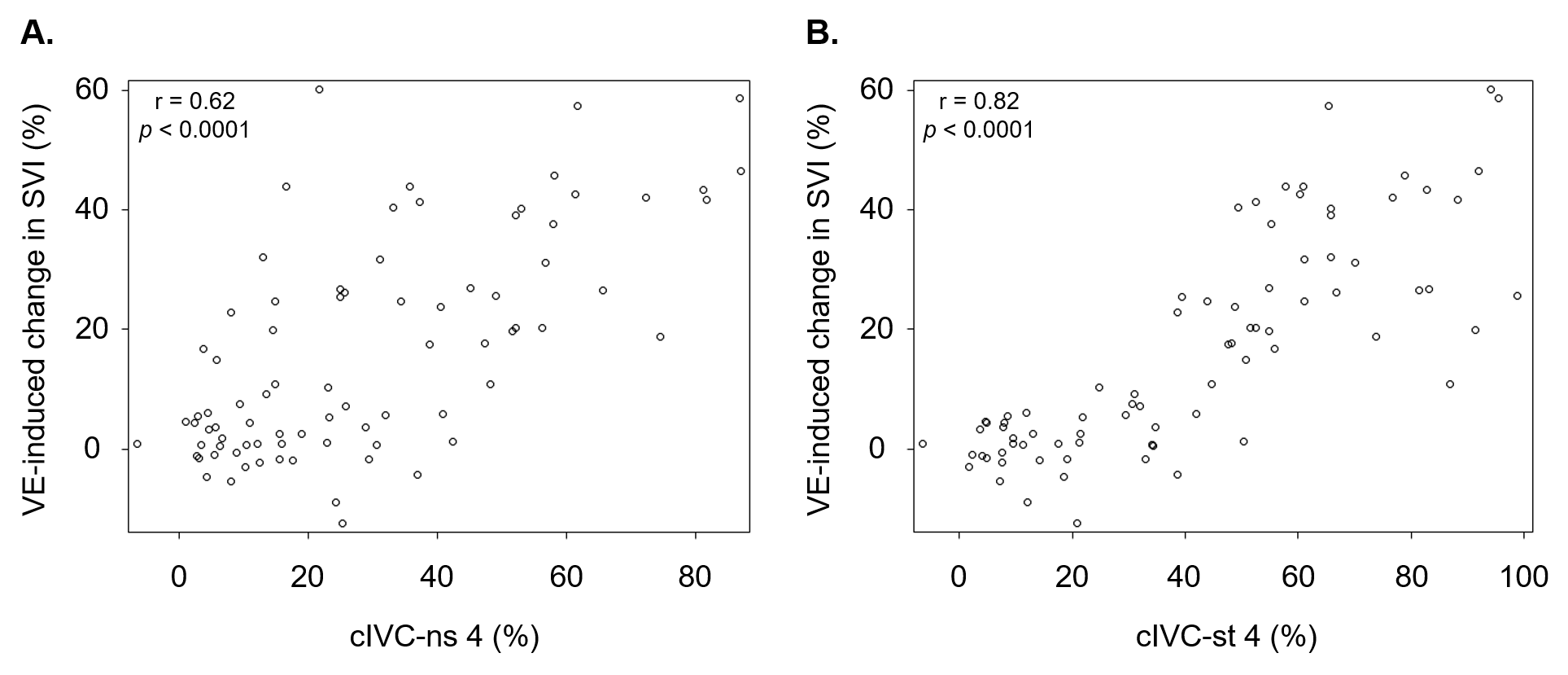

Supplement: Supplementary file 2 — Additional file 2. Linear correlation between the collapsibility index of the inferior vena cava (cIVC) and fluid responsiveness. [file 13613_2020_786_MOESM2_ESM.tif]
